# Feasibility Pilot: Problem Adaptation Therapy for Emotion Regulation in Community-Dwelling Older Adults

**DOI:** 10.1093/geroni/igab046.1585

**Published:** 2021-12-17

**Authors:** Julia Sheffler, Melissa Meynadasy, Dimitris Kiosses, Natalie Sachs-Ericsson

**Affiliations:** 1 Florida State University College of Medicine, Tallahassee, Florida, United States; 2 Florida State University, Tallahassee, Florida, United States; 3 Weill-Cornell Medicine, White Plains, New York, United States

## Abstract

Emotion regulation (ER) difficulties in older adults are associated with increased depression and decreased resiliency to stressful life events. In general, maladaptive ER is a transdiagnostic risk factor for a range of psychological and physical problems across the lifespan. Thus, interventions targeting ER may be valuable in reducing risk for a range of late-life pathologies. The present study evaluated and adapted an existing ER-focused treatment (i.e., Problem Adaptation Therapy (PATH)) for community older adults. We completed a small clinical pilot study to assess the feasibility of the adapted protocol and initial signals of effect of the intervention on ER, depression, and resiliency. Participants were recruited using an online survey, which was used to then identify participants scoring in the highest and lowest quartiles for ER. Individuals in the lowest ER quartile (N=27) were randomly assigned to the PATH condition or a physical health education (PHET) control condition. Of the 27 participants in the low ER group, four participants (3 PATH, 1 PHET) dropped out of the intervention. A paired samples t-tests revealed significant decreases in depressive symptoms, significant increases in self-reported ER skill, and improvements in resiliency (all ps<.05) for the PATH condition. For the PHET condition, only significant increases in self-reported ER skill (t(12) = -2.68, p = .020) were observed. In sum, the intervention protocol proved feasibility and demonstrated initial signals of effect in the expected directions. Future studies will examine mechanisms of action and the efficacy of the adapted PATH protocol.

